# Epigenetic Influences in the Aetiology of Cancers Arising from Breast and Prostate: A Hypothesised Transgenerational Evolution in Chromatin Accessibility

**DOI:** 10.1155/2013/624794

**Published:** 2013-02-03

**Authors:** Francis L. Martin

**Affiliations:** Centre for Biophotonics, Lancaster Environment Centre, Lancaster University, Bailrigg, Lancaster LA1 4YQ, UK

## Abstract

Epidemiological studies have consistently supported the notion that environmental and/or dietary factors play a central role in the aetiology of cancers of the breast and prostate. However, for more than five decades investigators have failed to identify a single cause-and-effect factor, which could be implicated; identification of a causative entity would allow the implementation of an intervention strategy in at-risk populations. This suggests a more complex pathoaetiology for these cancer sites, compared to others. When one examines the increases or decreases in incidence of specific cancers amongst migrant populations, it is notable that disease arising in colon or stomach requires one or at most two generations to exhibit a change in incidence to match that of high-incidence regions, whereas for breast or prostate cancer, at least three generations are required. This generational threshold could suggest a requirement for nonmutation-driven epigenetic alterations in the F0/F1 generations (parental/offspring adopting a more westernized lifestyle), which then predisposes the inherited genome of subsequent generations to mutagenic/genotoxic alterations leading to the development of sporadic cancer in these target sites. As such, individual susceptibility to carcinogen insult would not be based *per se* on polymorphisms in activating/detoxifying/repair enzymes, but on elevated accessibility of crucial target genes (e.g., oncogenes, tumour suppressor genes) or hotspots therein to mutation events. This could be termed a genomic susceptibility organizational structure (SOS). Several exposures including alcohol and heavy metals are epigens (i.e., modifiers of the epigenome), whereas others are mutagenic/genotoxic, for example, heterocyclic aromatic amines; humans are continuously and variously exposed to mixtures of these agents. Within such a transgenerational multistage model of cancer development, determining the interaction between epigenetic modification to generate a genomic SOS and genotoxic insult will facilitate a new level of understanding in the aetiology of cancer.

## 1. Introduction

**Figure 1:**
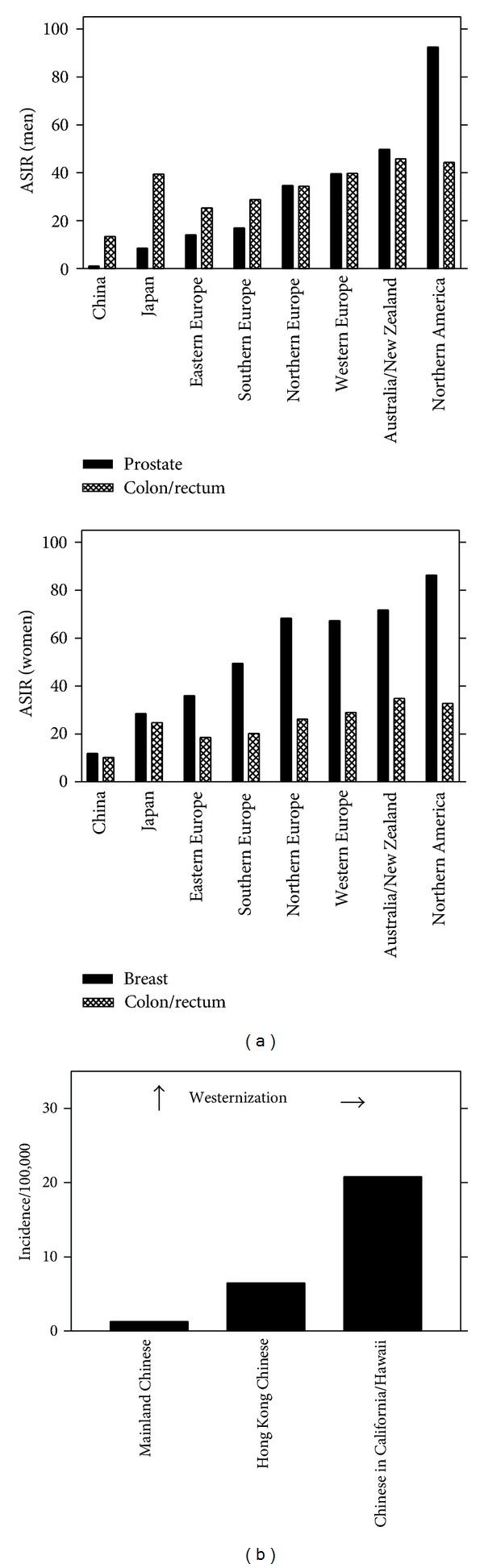
Incidence by region of breast, prostate, and colorectal cancers, and increase in prostate cancer in Chinese migrants. (a) Age-standardized incidence rate (ASIR) as estimated by Parkin et al. (1999) [2]. (b) Increasing incidence of prostate amongst Chinese migrants as estimated from Muir et al. (1991) [3].

Epidemiological studies clearly implicate environmental and/or lifestyle factors in the aetiology of cancers arising in hormone-responsive tissues, such as those from the breast or prostate [1]. This is based on the observations that incidence of these cancers is high in regions such as Northern/Western Europe and the USA, whereas recorded levels in other areas including China and India are traditionally some 10-fold lower [2] (Figure 1(a)). However, when populations migrate from these areas of low risk to high-risk regions, subsequent generations exhibit a disease incidence more in keeping with that of the host population [3, 4] (Figure 1(b)). Even amongst families identified with highly penetrant predisposing mutations in genes such as *BRCA1/2* and resident in low-risk areas, there appears to be a lower incidence compared to similar familial lineages resident in a westernized environment [5]. These observations begin to lay the basis of a complex and maybe transgenerational model of cancer induction in some hormone-responsive tissues.

 Within migrant populations, the cancer-incidence profile changes [4], but not at the same rate for all tissue sites. The incidence of colorectal cancer rises and that of stomach cancer falls in migrant populations from Far East areas more quickly and within two generations compared to the three generations required to observe similar increases in breast and prostate cancer [1]. This suggests an additional requirement to the simple initiation-promotion model of cancer development [6, 7]. The notion of a transgenerational requirement in cancer induction is not new: albeit intrauterine exposure occurred, diethylstilbestrol (DES) gave rise to marked increases in the unusual entities of adenosis and clear-cell adenocarcinoma of the genital tract in young female daughters of mothers exposed to this agent [8]; whether there were consequences in male offspring remains to be ascertained. More recently, models such as the Agouti mouse have shown that transgenerational influences can result in offspring predisposed to a pathological state such as obesity [9], in itself a grave predisposing factor for chronic morbidities.

 With more populations globally adopting a westernized lifestyle (that which could be associated with living in Northern/Western Europe and USA), there is the real possibility for a sudden surge in cancers of the breast, colon, prostate, and uterus in areas that hitherto would not have seen large rates of incidence of these conditions; in many of these regions, the question will be whether there will be a healthcare infrastructure capable of coping with markedly increased numbers of cases and, capable of providing appropriate treatment and after-patient care [10]. As far back as the 1960's, there was recognition of worldwide region-specific differences in breast cancer incidence and, correlations between calorie, protein or fat consumption, and risk were noted [11]. Even then, in addition to environmental carcinogen exposures, a hormone-mediated difference in susceptibility to breast cancer among US-resident women of different ethnic backgrounds was observed. Breast adipose tissue may act as a reservoir for lipophilic genotoxic carcinogens [12, 13], but still there is likely a requirement for a hormone-driven event [11] that develops or evolves with a phenotypic change associated with lifestyle.

 Diet predisposes children from ethnically diverse populations resident in the USA to chronic conditions such as cancer later in life; this same early-life influence is noted in the UK [14, 15]. The question then is whether due to differing environmental and/or lifestyle changes, a phenotypic change occurs in migrants to Western regions (e.g., Northern/Western Europe and USA) and their children (i.e., the F0/F1 generations), and this then not so much enhances vulnerability as a mutation might, but creates accessibility to critical target sites (e.g., tumour suppressor genes (TSGs) and oncogenes) in the genome of subsequent generations (Table 1). As a consequence the F2/F3/and so forth generations inherit a structurally vulnerable genome; this may be to allow increased growth and physicality associated with Western lifestyle, but it could also open up the potential for genomic accessibility with consequent cycles of chemical-DNA adduct formation and potentially faulty repair. This parental priming of the genome template could be termed a genomic susceptibility organizational structure (SOS); within this model, later generations do not inherit predisposing mutations but a chromatin organization that allows critical genes to be better targeted by DNA-damaging agents of environmental and/or dietary origin.

 Such developmental plasticity could be associated with epigenetic modifications. Environmental and/or lifestyle factors alter the epigenome of the F0/F1 generation amongst a migrant population to a high-risk region; these inherited epigenetic alterations would then predispose the genome of subsequent generations. This hypothesised model would explain why epidemiological studies to date have failed to identify causative factors for breast or prostate cancers although migration, hormone factors, calorie/protein/fat consumption, and exposure to carcinogens have been variously implicated. This review sets out to delineate a potential role for transgenerational epigenetic influences in the aetiology of breast and prostate cancer, cancers arising from these sites being more complex than more directly targeted tissues such as the lung, colon or stomach. It will also highlight that conventional epidemiological studies that typically set out to establish cause (or susceptible genotype) and effect, will be severely limited in their scope under this paradigm. However, it also shows why with ever increasingly large studies more robust associations with environmental causes are noted.

## 2. Epigenetic Alterations Underpinning Disease

Epigenetic alterations include modifications in DNA methylation, posttranslational influences on histone morphology/code (i.e., proteins forming the nucleosome core of chromatin and pivotal in structural packaging of DNA), nucleosome positioning and, the profile of microRNAs (miRNAs) and noncoding RNAs [16–18]. The most-studied alteration to date is methylation at the 5′ position of cytosines mediated by DNA methyltransferases (DNMT), predominantly in cytosine-guanine dinucleotide (CpG) sites; this generates 5-methylcytosine (5-meC), and if it occurs at sites that occupy the promoter regions of genes, this hypermethylation is correlated with transcriptional silencing.

 Methylation can result in complete transcriptional repression of TSGs such as *p16* [19]; if detected early enough, the specificity of this methylation pattern could be predictive for cancer types occurring at a particular or different sites [20, 21]. This has led to the development of quantitative methylation-specific PCR analyses to determine the methylation status of a panel of candidate TSGs in order to determine if one can triage for certain cancers [22]. Such methylation patterns may throw up new differentially expressed gene candidates in cancer, some of which may even be free of mutations [23]. These observations herald the possibility of epigenetic blood-based biomarkers linked to the underlying mechanism of a specific cancer pathogenesis [24]; it opens up possibilities of novel early screening tools.

**Figure 2:**
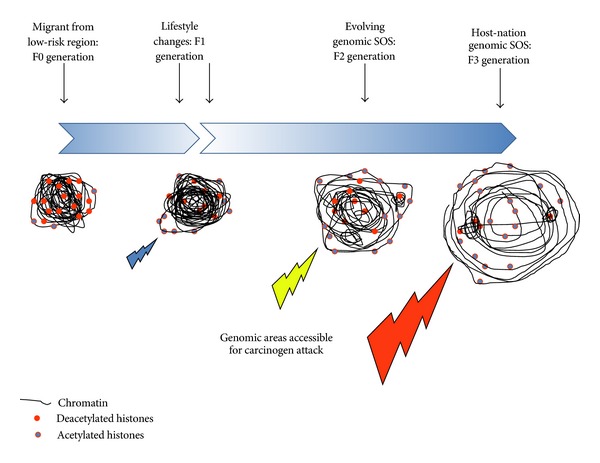
Transgenerational dependency of the genomic susceptibility organizational structure (SOS). Migrants (F0 generation) from a low-risk region for breast or prostate cancer exhibit a chromatin conformation that makes target genes often targeted for damage by environmental and/or dietary constituents less accessible. With lifestyle changes, a more accessible genome evolves in the F1 generation, as evidenced by a less compact chromatin and more acetylated histones; this creates more target sites for attack by carcinogens. Through F2, the genomic SOS further evolves, creating even more accessible sites for carcinogen attack. By F3, an evolution to more closely match that of the host nation genomic SOS has ocurred, with a maximum number of target sites for carcinogen attack. The break in the temporal arrow highlights a change of environment and/or lifestyle that leads to the generation of the genomic SOS; the increase in shade indicates its evolution.

 Although methylation status is currently the most-studied epigenetic marker, there is increasing recognition that other modifications such as those of the histone code can modify the chromatin organization in such a way as to influence in a dominant fashion regional mutation-rate [25]; this could be a pivotal observation to be translated to the notion of the evolution of a genomic SOS in humans. Histone deacetylases are now recognized to play a role in the control of the DNA damage responses at several levels [26]. What is compelling is that these structural chromatin alterations can be inherited [27]; this remodelled genome must influence the offspring's susceptibility to damage events following carcinogen exposures in their lifetime, that is, a genomic SOS. Metabolic signals play a critical role in determining chromatin structure [28]; thus one can surmise that given a markedly different lifestyle, the same genotype could generate a markedly different phenotype through the evolution of differing profiles of epigenetic marking of the genome. When migrants arrive in a new region, an initially slowly-adopted diet and/or lifestyle will then impact on the organizational structure and relative compaction of their offspring's chromatin (Figure 2). Migrants from the Far East to Northern/Western Europe may see their children/grandchildren grow more than those with similar genotypes in previous generations; this may be looked upon as a beneficial health outcome, but does this have an impact on the accessibility of their genome to genotoxic exposures of dietary and/or environmental origin? Studies of evolution have suggested that epigenetic drivers have underpinned dramatic changes in developmental biology, an example being that of cognitive function, to a far greater extent than genetic variability [29]. Persistent physical exercise will modify the epigenome, which will alter phenotype [30]; likewise, diet and/or lifestyle will undoubtedly play a significant role in this process too.

 Of additional recent interest is the role noncoding RNAs play in the pathogenesis of cancer. Although their biology and function still remain mostly obscure, they are believed to have core functions in a wide range of cellular processes, through interaction with key component proteins in the gene regulatory system. Alterations of their cell- or tissue-specific expression and/or their primary or secondary structures are thought to promote cell proliferation, invasion, and metastasis [31]. miRNAs comprise a family of small, endogenous, noncoding functional RNA molecules that have emerged as key posttranscriptional regulators of gene expression; they may again be useful biomarkers for early detection of disease-related molecular and genetic changes [32]. Noncoding miRNAs can contribute to cancer development and progression, and are differentially expressed in normal tissues compared to cancers; their expression signature may define cancer gene targets [33] or be diagnostic of the cancer type. However, not dissimilar to histone modifications, the expression profile of noncoding miRNAs constitutes an epigenetic alteration that seems to regulate male gamete production [34], CNS functions [35], cell cycle and proliferation [36], and erythrocyte development during haematopoiesis in vertebrates [37], amongst a plethora of other biological functions and mechanisms. In a mouse model, paternal exposure to the procarcinogen benzo[*a*]pyrene (B[*a*]P) affected the expression of several miRNAs, and the target genes for some of the dysregulated miRNAs were enriched in many different pathways that are likely to be relevant for the developing embryo [38]. The notion of an expression control by a posttranslational genomic signature generated by a previous generation's environmental influence, but governing the offspring's susceptibility, will pose extreme challenges for future epidemiological studies. This does not represent a simple cause-and-effect model within the individual but one modulated by ancestral environmental influences.

### 2.1. Lifestyle Factors That Modify the Epigenome

Early-life (pre- and/or postnatal) influences on the epigenome appear to modify risk to later-life susceptibility to chronic diseases, including cardiovascular disease, diabetes, and cancer [39]. In a number of animal models, the maternal nutritional environment appears to play a pivotal role in the long-term wellbeing of the offspring [40]; we are only now beginning to investigate how this might influence the epidemiology of chronic age-related diseases such as cancer in humans. Cancer is a heterogeneous disease, which we understand now is not only caused by genetic alterations (e.g., mutations) but also by an altered gene expression profile (i.e., epigenetic alteration) [41]. Given that we have known for years about a constitutive role for extrahepatic bioactivating enzymes capable of activating procarcinogens [42, 43], differences in expression between different regions or cell populations within the same tissue [44], and their environmentally-/lifestyle-mediated modifiable nature [45], the notion that epigenetic influences may also modify their role in cancer aetiology is not unsurprising. Differences in environmental influences mean that older monozygous twins who may have grown up in differing environments (e.g., urban *versus* rural) exhibit significant discordance in epigenomic markers such as genomic distribution of 5-meC DNA and histone acetylation compared to those examined in the early years of life [46]. Epigenetic changes in DNA methylation patterns at CpG sites (which could be known as epimutations) may give rise to transgenerational effects [47], although understanding these phenomena in real-world situations remains a major challenge [48]. However, the premise of this review is that a transgenerational step is required towards acquiring the elevated risk of breast or prostate cancer observed in North/Western Europe and the USA. This differs from a more cause-and-effect model seen with lung cancer induced by tobacco smoke, stomach cancer induced by nitrosamines [49], or hepatocellular carcinoma induced by aflatoxin B_1_ [50].

### 2.2. Could Epigens Modify Early-Life Risk? 

Ever since valproic acid was identified as a specific histone deacetylase (HDAC) inhibitor [51], it has also been speculated that new intervention or therapeutic strategies exploiting agents that also modify transcriptional regulation in the pathogenesis of cancer *via* the generation of epigenetic alterations could be developed. Although this may convey a novel therapeutic strategy in the presence of disease, it also raises the possibility that continuous low-level exposures to epimutagenic agents might enhance susceptibility to genotoxic mechanisms [52]. A whole range of chemical contaminants found in the human diet and/or environment may modify the epigenomic patterns of cytosine methylation and/or histone acetylation [53]. Examples of this are the heavy metals including arsenic, nickel, chromium, and cadmium, which increase cancer incidence; these are weak mutagens but appear to be potent modifiers of the epigenome [54]. By examining morphological changes in heterochromatin and DNA methylation at gene loci in an *in vitro* early embryo (mouse) model, it was shown that selected environmental contaminants, including diethyl phosphate, mercury, cotinine, selenium, and octachlorodipropyl ether induced at low concentrations (i.e., ppb), marked and sometimes irreversible epigenetic alterations [55]; given the potential for real-world exposures to such exogenous agents, one might surmise that this could impact human embryological development. Of course, humans are not exposed to single test agents but are exposed continuously and variously throughout life to a changing cocktail of different contaminants [56]. Modelling the potential effects of such mixtures is a challenge, as different test agents might be similarly acting or independently acting [57, 58]; as such, they could act in combination in additive, synergistic, or inhibitory mechanisms. In populations with high exposures to persistent organic pollutants (POPs), such as Greenlandic Inuit, global methylation levels were inversely associated with blood plasma levels for several POPs [59]. Such observations make it highly likely that epigens (i.e., modifiers of the epigenome) found routinely in the human diet and/or environment could modify the human epigenome, which could add another level of complexity in the exposure-causation model of classical epidemiological studies [60]. If these heritable genomic alterations are a consequence of parental or early-life exposures, they may if they occur greatly enhance subsequent susceptibility to later-life factors in the aetiology of chronic disease.

## 3. Chemicals That Modify the Epigenome

There is an increasing suggestion in the academic literature that exposure to environmental contaminants may play an aetiological role in a range of disease-predisposing conditions, including obesity. Although high-density calorie diet and lack of physical activity might be the primary causes of obesity, endocrine disruptors acting as obesogens could initiate or exacerbate this morbidity [61, 62]. In addition to endocrine disruptors, there is a growing body of evidence that heavy metals, including nickel, lead, cadmium, arsenic and others, asbestos, and alcohol intake all act to variously modify the potential for epigenetic alterations [63]. Air pollution constituents, especially particulate matter (PM), appear to alter the profile of miRNAs [18]. PM is known to alter epigenetic markers (e.g., DNA methylation and histone modifications), which may contribute to air-pollution-mediated health consequences including an elevated risk for cardiovascular diseases or events; identifying individual epigenetic loci associated with dysregulated gene expression following exposure could generate novel intervention strategies mitigating the development of such adverse outcomes [64]. Ionizing radiation and nanomaterials are also thought to induce epigenetic alterations. In addition, perfluorinated compounds are of significant concern as they bioaccumulate with suggestions that *in utero* human exposure is associated with global hypomethylation of the genome; recently, perfluorooctanoic acid-mediated toxicity was associated with aberrant methylation of glutathione-*S*-transferase Pi [65], a carcinogen-detoxifying enzyme.

 Chemical pollutants, dietary components, temperature changes, and other external stresses can indeed have long-lasting effects on development, metabolism, and health, sometimes even in generations subsequent to the exposed individual [66]. A growing body of epidemiological evidence demonstrates associations between parental usage (especially occupational) of pesticides, particularly insecticides, giving rise to acute lymphocytic leukaemia and brain tumours in offspring [67]. Accumulating evidence suggests that environmental and occupational exposures to natural substances, as well as man-made chemical and physical agents, play an aetiological role in human cancer; carcinogenesis may be induced by either genotoxic or nongenotoxic carcinogens (e.g., arsenic, 1,3-butadiene) that also cause prominent epigenetic changes [68]. Cadmium is a toxic, nonessential transition metal and contributes a health risk to humans, including strong associations between its exposure and various cancers or cardiovascular diseases. This agent has been shown to induce various epigenetic changes in plant and mammalian cells *in vitro* and *in vivo*, and this is likely the primary mechanism *via* which it mediates its toxicity [69].

 The importance of early-life changes towards future susceptibility to chronic age-related diseases is gaining increasing recognition. Of major concern in this regard are observations that common environmental contaminants such a bisphenol A and phthalates can variously be hypomethylating and alter miRNA expression levels or DNMT activities [70]; the fact that such agents appear to induce low-dose effects postmaternal exposure in the genital tract of female offspring of mice [71] suggests a phenotype change associated with an epigenetic alteration that has later-life consequences. In fact, in the area of environmental epigenetics, such agents as well as other endocrine disruptors including organochlorines, are likely to play a pivotal role [72]. This could be an important link with cancers arising from hormone-responsive tissues, including the breast and prostate. Disruptors of hormonal status via epimutagenic processes are yet to be understood in terms of their long-term health consequences.

The interplay between genotoxicity and epigenetic alterations remains to be elucidated; for instance, acetylation of histones occurs during the process of DNA damage induction [73]. As direct-acting DNA-damaging agents are traditionally known as genotoxins, agents that induce aberrant epigenetic alterations may be known as epimutagens. In general, cancer is typified by global genomic hypomethylation and site-specific hypermethylation [74], especially at TSGs: the former being associated with an overactive genome and proliferation, and the latter with inactivation of genes such as TP53 that might sit at the crossroads between induction of aberrant proliferation and apoptosis. Time of exposure during life, dose, gender, and organ specificity all need to be considered in the development of epigenetic endpoints as biomarkers for exposure to epimutagenic toxicants [75]. How at different stages of life in a particular target organ there is induction of irreversible changes to the genetic material (i.e., DNA mutations) against a backdrop of putatively reversible changes to the epigenetic landscape (i.e., changes in the DNA methylation and chromatin modification state) remains to be understood [76]. Does the latter modify accessibility of the genome in a fashion that predisposes it to genotoxic insult? This may underpin the interplay between genotoxic and epigenetic mechanisms in the aetiology of cancer.

## 4. Epidemiology and the Epigenome

Linking exposures and their possible effects to environmental health is a major inter-disciplinary challenge [77]. Inter-individual variability increases with age and is probably mediated by an environmental modification of the epigenome; this could explain why genetically identical twins might age differently [78]. In fact, the ageing process has recently been observed to be associated with an increasing level of global genomic hypomethylation [79]. In lung cancer tissues, several gene loci were observed to be either significantly hypermethylated or hypomethylated [80]. Ideally, epidemiological studies require early biomarkers of risk so that susceptible individuals and/or populations can be identified. In theory, this would facilitate intervention studies within which damaging exposures would be reduced or removed [81]. Because epigenetic alterations are modifiable, this has enormous implications for cancer prevention and treatment [82].

 In an era of epidemiology that has been largely based on attempting to correlate gene-environment interactions to identify environmental causative factors imposed on a profile of genetic susceptibility [83], determining parental or ancestral lifestyle and/or environmental influences on transgenerational susceptibility to chronic diseases such as cancer will be a major challenge. Populations that migrate from regions of low disease incidence to high-risk areas do not just face changes in environment (which may in fact become more contaminant-free and regulated in the case of westernized regions), but primarily, and especially in subsequent generations, profound cultural and lifestyle changes. This has resulted amongst Asians migrating to the UK in a higher prevalence of Type II diabetes compared to the multigenerational resident population [84], obesity and coronary heart disease [85]. Within a laboratory model such as the Agouti mouse, one may generate interesting mechanistic paradigms of transgenerational effects. However, given that human lifestyle has changed so dramatically in the last few generations, especially in Northern/Western Europe and USA, factors that were either potentially deleterious (e.g., smoking) or even beneficial (e.g., high calorie storage; [86]) may manifest an increased susceptibility or protection in a subsequent generation. Understanding ancestral influences on current risk stratification in today's global population is going to be a major challenge in coming years.

### 4.1. Genotoxins/Mutagens versus Epimutagens in Cancer Aetiology

The traditional model of cancer causation is based on an initiating exposure (independent of age) by a genotoxic/mutagenic agent followed by a multistage process of promotion, which may take >20 yrs in humans [87, 88]; invasive disease characterized by gross genomic instability then progresses beyond this. In the absence of the inheritance of highly-penetrant mutant alleles (e.g., *BRCA1/2*), this model has laid the basis for our understanding of the aetiology of sporadic cancers. The need for an initiating exposure was first proposed by Roger Case in his studies of bladder cancer incidence in dye industry workers [89, 90], and subsequent studies of the role of cigarette smoking in lung cancer [91, 92] have been pivotal in the acceptance of this theory. Molecular archaeology studies clearly demonstrate that a specific environmental/dietary chemical exposure (e.g., aflatoxin B_1_) can induce a particular mutation in localized “hotspots” of critical genes (e.g., the tumour suppressor *TP53*) in hepatocellular carcinoma [93, 94].

 One can now point to several types and classes of physical and chemical environmental and dietary carcinogens, which will with or without a bioactivation step (the latter required in the case of inert chemicals) induce a genotoxic event, ultimately giving rise to mutations in target cells. These include polycyclic aromatic hydrocarbons (PAHs) such as B[*a*]P [95], heterocyclic aromatic amines (HAAs) such as 2-amino-1-methyl-6-phenylimidazo [4,5-*b*]pyridine (PhIP) [96], physical agents such as ultraviolet (UV) or ionizing radiation [97], and materials including asbestos fibres [98, 99]. With indirect or inert chemical carcinogens, a bioactivation step is required (mediated primarily by cytochrome P450 mixed function oxidases (CYPs), but also by *N*-acetyltransferases (NATs) or sulfotransferases) to generate electrophilic intermediates that then give rise to covalent DNA adducts at nucleophilic sites on DNA bases, for example, C8 position of deoxyguanosine [100, 101]. The generation of base substitution or frameshift mutations then occurs *via* the inaccurate repair of the adducted template, which has been associated with the replication complex performing a mutagenic bypass of the lesion by a slippage mechanism [102, 103]. The question is how does the epigen determine the magnitude of the mutagen or genotoxin effect?

Chromatin accessibility plays a role in not only regulating cell-type specific gene expression [104], but also DNA repair [105]; if epigenetic mechanisms determine the accessibility of pivotal genes to electrophilic attack giving rise to DNA adducts and to subsequent faulty repair mechanisms, this might be a plausible link between the genotoxic insult and the epigenomic modification governing individual susceptibility to cancer causation. The transgenerational mechanism is that the epigenomic modification was inherited. As such, the offspring does not have an inherently susceptible genome in terms of polymorphic differences in a panel of susceptibility genes (e.g., bioactivating, DNA repair, or detoxification), but the structural dimensions of the chromatin facilitate its vulnerability to exogenously- and/or endogenously-derived insults. Thus the parental or ancestral environment and/or lifestyle generated the evolution of a genomic SOS, that is heritable, and predetermines from early life the offspring's susceptibility to genotoxic carcinogens. Within the accessible genome, there is a greatly elevated chance of the initiation-promotion multistage process of carcinogenesis occurring.

### 4.2. Factoring Transgenerational Epigenetic Modifications into Future Epidemiological Studies

Before embarking on an epidemiological study, sample size calculations are important to provide evidence that the proposed study is capable of detecting real associations between study factors [106]. Traditionally, to know what size of relative risk may be confidently detected with the projected size of the cohort and length of followup, one often determines the adequacy of cohort size at the planning stage of a study [107]. Disease epidemiology aiming at identification of carcinogens and quantification of associated risks has always had an apparent low resolving power, with detectable incidence or mortality increments often being orders of magnitude smaller than levels which would be of public concern. Other drawbacks of disease epidemiology are the long latency times in development of chronic diseases, difficulties in reliably tracking large population cohorts and the influence of confounders [108]. Biomarkers such as gene polymorphisms or chemical-DNA adducts were through the 1990's employed as measures to improve cause specificity [108–110]. In the main, these have proved limited in their ability to generate robust risk associations. With the increasing development of systems biology approaches that generate large and complex datasets, deriving significant associations with chronic conditions such as cancer will become more challenging [111]. For instance, in genome-wide association studies (GWAS) that aim to identify genetic variants related to diseases by examining the associations between phenotypes and hundreds of thousands of geno-typed markers, new theoretical frameworks are required [112].

 Chemical-DNA adducts do occur in target tissues such as the prostate, although their levels may not correlate with expression levels of bioactivating enzymes [113]. To estimate sample size using a simple cause-and-effect model is difficult. The majority of studies have failed to provide robust associations with common complex diseases; that said increasing sample size dramatically may be capable of achieving this, and these observations have given stimulus to the implementation of biobank studies worldwide [114]. However, incorporation of a transgenerational component into epidemiology studies could require the incorporation of an uncertainty factor in their design. This could also explain why exceedingly large studies are required to robustly isolate cause and effect against the major confounder of inherited chromatin organization determined by ancestral, environmental, and/or lifestyle factors.

## 5. Nurturing the Epigenome

The complex interplay between nature (could be understood as genotype) and nurture (understood as lifestyle and/or environment) has been considered to hamper efforts to specify quantitatively the relative contribution of either to disease causation [115]. Questions traditionally addressed would be whether particular polymorphisms in key genes (e.g., *NAT2*) could be associated with a disease endpoint (e.g., bladder cancer) in Chinese subjects exposed to an agent (e.g., benzidine); here, there appears to be a protective effect of the slow acetylator genotype [116]. Although individual combinations of polymorphic traits may modify susceptibility to exposures [117], in the complex disease scenario many studies have failed to show robust risk associations with genotype [118–120]. Spontaneous deamination of 5-meC to thymidine (which is not excised or repaired) could be a significant source of signature mutations [121] in particular cancers. These C→T transitions at CpG sites appear to be prevalent mutations in *TP53* in human colon cancer [122]. In the context of this hypothesis, the question might be whether the definition of an epimutagen could be expanded not just to include altering the methylome, histone code, or profile of noncoding RNAs, but also the altering of the accessibility of important regions of the chromatin to genotoxic insult.

 As lifetime exposures to environmental carcinogens might be expected to increase risk, especially in genotypes perceived as vulnerable, for example, glutathione-*S*-transferase null [123], other lifestyle factors such as consumption of fruit and vegetables appear to be protective against risk of disease [124, 125] and even initial DNA damage [126]. However, with the emergence of the epigenomics field the role of modifiable factors takes on another dimension. One could surmise under the model hypothesized here that environmental and/or lifestyle factors typically associated with a Western region might induce the evolution of a genomic SOS. However, are there risk factors such as tobacco smoke or infection that potentiate or accelerate this and thus greatly increase the accessibility of chromatin in future generations to genotoxic insult? Conversely, are there protective factors such as healthy intakes of fresh fruit and vegetables that counteract these effects and retard the evolution of the genomic SOS? Within epidemiology, deciphering the role of transgenerational influences mediated by epigenetic marking of the chromatin may require a detailed human epigenome project [127–129]. This could allow a stratification of predisposing early-life risk based on the structural organization of the inherited genome and the expression pattern therein. Given the even more individualized nature of the epigenome compared to the genome (it being the genome modified by environment), the complexity of this task could take several years to unravel [130].

 Within this context it will be interesting to determine whether the epigenome of an individual is relatively similar across all tissues or whether there are between-organ and within-organ differences in the epigenomic profile. For years there has been evidence of a field effect in cancer in which the surrounding tissue might provide the microenvironment allowing the cancerous growth. This has been associated with nuclear changes (possibly epigenetic) and downstream epigenetic-associated events including changes in gene and protein expression, DNA damage, and angiogenesis [131]. Thus in prostate, the androgen receptor promotes growth of cancer initiating cells *via* autonomous signalling pathways, whereas there is a lack and no apparent need for androgen receptor signalling in surrounding stroma [132]. The question would be whether there are differing epigenomic profiles even within the same tissue, some which may generate a field change necessary to nurture the promotion and progression of disease, and others necessary for the clonal expansion of an initiated cell(s). Additionally, there is emerging interest in exosomes, which are extracellular subnanosized vesicles that are believed to contain defined patterns of mRNA, miRNA, noncoding RNA, and occasionally genomic DNA [133]. This may be a relatively unexplored mechanism of intercellular communication in which transferred genetic information may induce transient or persistent phenotypic changes in recipient cells. Exosomes play a fundamental biological role in the regulation of normal physiological as well as aberrant pathological processes, *via* altered gene regulatory networks and/or *via* epigenetic programming [134]; how they may modulate the microenvironmental epigenome remains to be ascertained.

In the past, correlations of environmental exposure and cytogenetic biomarkers have been very complex [135] because of the interaction of independent factors (e.g., genotype *versus* exposure). Often within each factor, there will be a requirement to stratify for variables such as age or gender. Ancestral influences on the epigenome could be yet another such variable. How such a variable could be readily factored into large-scale epidemiological studies is uncertain. In general one relies on biological material that is readily available, including blood (whole serum or plasma), buccal mucosa, or urine. Circulating free DNA in plasma has often been used [136]. Whether epigenetic alterations in surrogate tissues such as peripheral blood lymphocytes correlate with exposures (ancestral *versus* present) and can reflect disease risk or development in a target tissue remains to be determined.

### 5.1. Consequences for Breast and Prostate Cancer Incidence in Traditionally Low-Incidence Regions

As more regions become increasingly westernized in their lifestyle, based on the genomic SOS model, one could surmise that the environmental and/or dietary influences are already occurring in the present generation in traditionally low-risk regions of China, India, or Japan; this will predispose the next generations to higher incidences of breast and prostate cancer. Figure 1(a) shows that incidence of colorectal cancer in Japan is now not dissimilar to levels observed in Northern/Western Europe or the USA, the former being traditionally a low-risk region for this disease. However, levels of breast or prostate cancer still remain relatively low in this region; that said, are epigenetic modifications already occurring that in the next decades will predispose this population to markedly higher levels of this disease? This transgenerational switch in chromatin accessibility could be a dichotomous marker to the offspring's commitment to an elevated risk. The development of robust and high-throughput sensor platforms examining the *in situ* methylation status or genomic SOS may be far off [137].

## 6. Conclusions

Despite an overwhelming amount of epidemiological evidence pointing to a role for environment and/or lifestyle in the aetiology of breast and prostate cancer, the failure to identify causative factors has led some to question this in favour of endogenous entities, for example, reactive oxygen species and hormones [138]. Chronic inflammation has been highlighted as a pivotal endogenous cancer-predisposing factor [139]. However, how ancestral exposures may influence the accessibility and consequent susceptibility of an individual's genome remains to be understood. This transgenerational influence may not be applicable in all cancers (e.g., lung, colorectal, or stomach) in which there might be more direct applications of the causative agent(s) (e.g., tobacco smoke constituents, HAAs, or nitrosamines) to the target site. As a consequence, such cancers exhibit a faster rate of increasing or decreasing incidence in migrant populations from low-/high-risk regions [4].

 The additional generation gap between migration from a low-risk region and attaining an incidence of breast or prostate cancer similar to the host populations points to a transgenerational step. The hypothesis laid out herein is that in the F0/F1 generations there occurs an evolution towards a genomic SOS, a model within which the chromatin becomes more accessible for genotoxic insult (Figure 2). This organizational structure of the chromatin is inherited and could be considered an epimutagenic event lending increased susceptibility to the organism and preceding mutagenic events incurred *via* environment and/or lifestyle factors by the F2/F3 generations. Such a model could partially explain why traditional epidemiological studies have in the main failed to draw firm cause-and-effect conclusions. A combination of novel sensor technologies and epigenetic biomarkers will be needed to order to integrate ancestral epigenetic influences at the organism, organ, and within-organ levels into future studies designed to give insights into the aetiology of these cancers. If ancestral influences such as environment and/or lifestyle markedly influence susceptibility to chronic diseases such as sporadic breast or prostate cancer, this will have enormous social as well as biological implications.

## Figures and Tables

**Table 1:** How a genomic susceptibility organizational structure (SOS) may influence progeny responses to environmental exposures.

Generation	Evolution of genomic SOS	
	Chromatin modification	Cancer risk
F0	−	Low
F1	+	Low
F2	++	Emerging
F3	+++	High

Translation of epigenetic alterations amongst migrant populations from low-risk cancer (breast and prostate) areas to high-risk regions. Parental generation has not acquired a genomic SOS, but by F1 and subsequent generations this organizational structure has emerged *via* environmental and/or lifestyle changes. This exposes the F2/F3 genomes to DNA damage insult.

−: not present; +: evolving; ++: evolved; +++: highly evolved.

## Abbreviations

PhIP: 2-Amino-1-methyl-6-phenylimidazo [4,5-*b*]pyridine
B[*a*]P: Benzo[*a*]pyrene
CYPs: Cytochrome P450 mixed function oxidases
CpG: Cytosine-guanine dinucleotide
DES: Diethylstilbestrol
DNMT: DNA methyltransferases
GWAS: Genome-wide association studies
HAAs: Heterocyclic aromatic hydrocarbons
HDAC: Histone deacetylase
miRNAs: MicroRNAs
5-meC: 5-methylcytosine
NAT: *N*-Acetyltransferase
PAHs: Polycyclic aromatic hydrocarbons
POPs: Persistent organic pollutants
SOS: Susceptibility organizational structure
TSGs: Tumour suppressor genes
UV: Ultraviolet.
